# CXCR3-independent role of CXCL10 in alveolar epithelial repair

**DOI:** 10.1152/ajplung.00301.2023

**Published:** 2024-05-21

**Authors:** Yanli Zhang, Jiurong Liang, Jun Ye, Ningshan Liu, Paul W. Noble, Dianhua Jiang

**Affiliations:** ^1^State Key Laboratory of Common Mechanism Research for Major Diseases, Institute of Basic Medical Sciences, Chinese Academy of Medical Sciences and Peking Union Medical College, Beijing, People’s Republic of China; ^2^Department of Medicine, Cedars-Sinai Medical Center, Los Angeles, California, United States

**Keywords:** alveolar repair, alveolar type II epithelial cells, CXCL10, TrkA

## Abstract

The alveolar type II epithelial cells (AEC2s) act as stem cells in the lung for alveolar epithelial maintenance and repair. Chemokine C-X-C motif chemokine 10 (CXCL10) is expressed in injured tissues, modulating multiple cellular functions. AEC2s, previously reported to release chemokines to recruit leukocytes, were found in our study to secrete CXCL10 after bleomycin injury. We found that Sftpc-Cxcl10 transgenic mice were protected from bleomycin injury. The transgenic mice showed an increase in the AEC2 population in the lung by flow cytometry analysis. Both endogenous and exogenous CXCL10 promoted the colony formation efficiency of AEC2s in a three-dimensional (3-D) organoid growth assay. We identified that the regenerative effect of CXCL10 was CXCR3 independent using *Cxcr3*-deficient mice, but it was related to the TrkA pathway. Binding experiments showed that CXCL10 interacted with TrkA directly and reversibly. This study demonstrates a previously unidentified AEC2 autocrine signaling of CXCL10 to promote their regeneration and proliferation, probably involving a CXCR3-independent TrkA pathway.

**NEW & NOTEWORTHY** CXCL10 may aid in lung injury recovery by promoting the proliferation of alveolar stem cells and using a distinct regulatory pathway from the classical one.

## Graphical abstract

**Figure:**
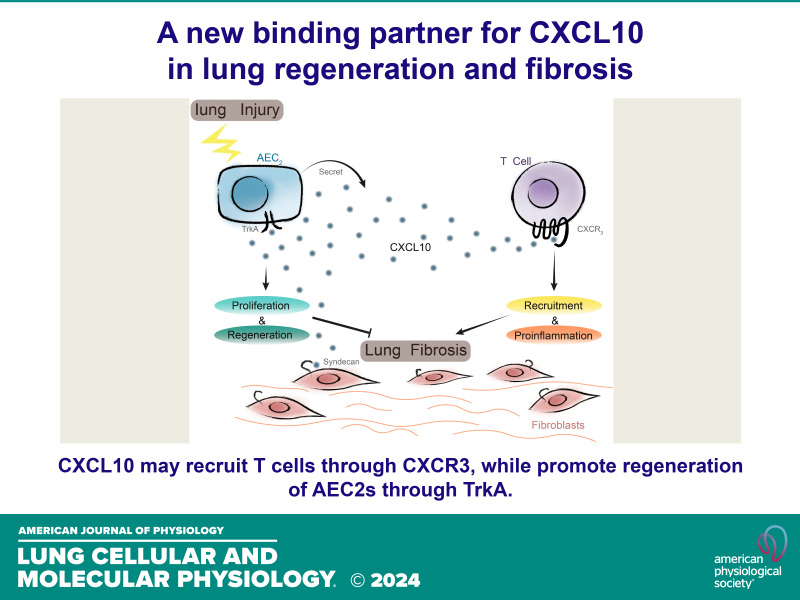

## INTRODUCTION

During lung injury and repair, chemokines are released from various cell types, such as epithelial cells, endothelial cells, fibroblasts, and leukocytes. They play a critical role in regulating injury and inflammation (1). The C-X-C motif chemokine 10, CXCL10, is upregulated in almost all tissues after infectious injury, noninfectious injury, or even trauma, suggesting an important role in the pathogenesis of tissue injury. Depending on the expression of different receptors, CXCL10 exerts different biological functions on different kinds of cells. CXCL10 mediates the effects of IFN-γ and induces chemoattraction of inflammatory cells mostly through its receptor CXCR3 on hematopoietic cell type (2, 3), and produces antiproliferative and antifibrotic effects through glycosaminoglycan proteins on endothelial and fibroblast cells (4, 5). However, the mechanism of CXCL10 on epithelial cell regeneration is largely unknown.

Alveoli are the gas exchange site of the lung, lined by type 2 and type 1 alveolar epithelial cells (AEC2s and AEC1s). Timely and orderly repair of the alveolar epithelium after injury is necessary to restore lung gas exchange function. AEC2s function as surfactant protein producer normally and have been described as alveolar stem/progenitor cells that can proliferate and differentiate into AEC1s (6–8). A lineage tracing study confirmed AEC2s to be stem cells in adult lung with the ability to self-renewal and differentiation over about a year (9). The study found that survivors of AEC2s experienced rapid clonal expansion and daughter cell dispersal when many AEC2s were specifically ablated. The regeneration of AEC2s demonstrates an important role in lung repair (9). We previously showed that there is a loss of AEC2s, and a failure of AEC2 renewal in idiopathic pulmonary fibrosis (IPF) (10). Stem cell regeneration and mobilization have been reported to be regulated by many cytokines and chemokines (1, 11, 12). The stromal cell-derived factor-1 α/C-X-C chemokine receptor 4 (SDF-1/CXCR4) axis regulates hematopoietic stem cell and progenitor mobilization, playing a role in tissue repair, regeneration, and development (13–15). IL-6 was found to promote regeneration of airway-ciliated cells from basal stem cells during the repair of adult tracheal epithelium post-SO_2_ injury (16).

The role of the CXCL10 chemokine on AEC2 regeneration has not been investigated, although a few studies have explored the expression of CXCL10 in lung epithelial cells (17). We hypothesized that CXCL10 plays a direct autocrine-like role in the regeneration of AEC2s. In the present study, we detected CXCL10 expression in injured AEC2s and examined the role of CXCL10 on AEC2 injury and repair in noninfectious lung injury using intratracheal instillation of bleomycin as a model of acute lung injury. We demonstrated that CXCL10 is critical in regulating lung AEC2 regeneration.

## MATERIALS AND METHODS

### Mice

Sftpc-Cxcl10 transgenic mice were generated by cloning the mouse *Cxcl10* cDNA downstream of the human Sftpc promoter and upstream of SV40 tAg polyadenylation. Transgenic mice were generated in C57Bl/6 F2 eggs using standard pronuclear injection at Duke University. Several lines of the CXCL10 transgenic mice were generated. Lines 2 and 3 were used in the study. The transgene was genotyped via PCR with the following transgene-specific primers: 5′-AAC TCA CCC AGG TTT GCT CTT-3′ (forward) and 5′-GGG CAA TTA GGA CTA GCC ATC-3′ (reverse). Scgb1a1 (Cc10)-Cxcl10 mice were described previously (18). Mice were housed and cared for in a pathogen-free facility at Duke University and Cedars Sinai Medical Center, and all animal experiments were approved by the Institutional Animal Care and Use Committee at Duke University and Cedars Sinai Medical Center.

### Bleomycin Lung Injury

Bleomycin was given to Sftpc-Cxcl10 mice and wild-type (WT) mice intratracheally. Mice were anesthetized and lungs were harvested for bronchoalveolar lavage (BAL) 0, 5, 7, or 10 days after injury.

### Bronchoalveolar Lavage

Bronchoalveolar lavage (BAL) was performed as described previously (19). Mice were anesthetized by 100 mg/kg ketamine and 10 mg/kg xylazine mixture and the lungs were perfused with PBS through right ventricle (10 mM, pH 7.4; 5 mL/mouse). The trachea was cannulated and the lungs lavaged three times with 0.8 mL aliquots of PBS. The live cells were recovered and counted with a hemocytometer.

### Bio-Plex and ELISA

Levels of mouse chemokine in the BAL or culture medium were measured with commercial ELISA kits (R&D Systems, Minneapolis, MN) or Mouse Cytokine Magnetic 20-Plex Panel kits (LMC0006M, Invitrogen, Thermo Fisher Scientific Inc., Waltham, MA) on Bio-Plex 200 system (Bio-Rad, Hercules, CA) according to the manufacturer’s instructions.

### Normal Two-Dimensional Cell Culture

Murine lung fibroblasts (MLgs; American Type Culture Collection, Manassas, VA) were cultured in MEM, supplemented with 10% FBS (Gibco), Non-Essential Amino Acids (NEAA), and split every 3 days. Sorted AEC2s (2 × 10^4^) were resuspended in a basic medium that contains Dulbecco’s modified Eagle’s medium-F12 (Gibco, Carlsbad, CA) supplemented with insulin-transferrin-selenium (Gibco), 10% FBS (Gibco), and cultured for 96 h, medium was collected every 24 h for ELISA detection of CXCL10.

### Matrigel Three-Dimensional Culture

The three-dimensional (3-D) culture of lung epithelial cells in Matrigel was as previously described (10, 20). In brief, sorted lung epithelial cells (3 × 10^4^ cells/mL) were mixed with mouse fibroblast MLg2908 cells (2 × 10^6^ cells/mL) in Matrigel/basic medium (1:1). Cells suspended in Matrigel/basic medium were added to the chamber of 24-well Transwell filter inserts (Becton Dickinson, Franklin Lakes, NJ,) and placed in 24-well, flat-bottom culture plates containing basic medium with 10 µM SB431542, with or without recombinant CXCL10 protein (R&D Systems, Minneapolis, MN), antibody to CXCL10 (R&D Systems), and antibody to CXCR3 (R&D Systems), isotype control (R&D Systems). Cultures were incubated at 37°C in a humidified incubator (5% CO_2_), and the medium was replaced every other day for up to 10 to 14 days. Colony-forming efficiency was determined by counting the number of colonies growing out.

### Histology

The mouse trachea was cannulated, and the lungs were inflated with 10% neutral buffered formalin (Sigma). The tissues were fixed overnight, embedded in paraffin, and sectioned (4 µm) for staining with trichrome.

### Hydroxyproline Assay

Collagen contents in lungs of 5–10 mice in each group were measured using a conventional hydroxyproline method (21).

### Histology and Immunofluorescent Staining

Cultured colonies were fixed with 4% paraformaldehyde in PBS overnight at room temperature. After rinsing with PBS, fixed colonies were immobilized with low melting agarose (Thermo Fisher Scientific Inc.) for embedding in paraffin. The colonies were sectioned (8 µm) and stained with antibodies against T1α, SPC (Millipore, Thermo Fisher Scientific Inc.), and mounted with ProLong gold antifade reagent with DAPI (Life technology). Colonies were visualized using a Zeiss LSM 780 inverted confocal microscope (Carl Zeiss MicroImaging Inc., Thornwood, NY).

### Flow Cytometry

Mice were anesthetized, perfused with PBS through right ventricle, and then the lungs were harvested. Lung cell suspensions were prepared for fluorescence-activated cell sorting (FACS) as described previously (10, 20). To obtain single-cell solutions, the trachea was cannulated, and the lungs were inflated with 1 mL of elastase (4 U/mL) and incubated for 30 min at 37°C water bath. An additional 2 mL of elastase was injected into lungs through trachea during incubation to keep the lung inflated. Then the lungs were chopped into small pieces and digested by 0.1 mg/mL of DNAse I in DMEM. A lysing buffer (eBioscience, San Diego, CA) was used to lyse red blood cells in the lung solution. Then cells were resuspended in Hanks’ balanced saline solution (HBSS) buffer supplemented with 2% fetal bovine serum (FBS), 10 mM HEPES, 0.2 mM EGTA, 100 IU/mL penicillin, 100 lg/mL streptomycin, and 0.25 lg/mL fungizone. For AEC2s sorting or analysis, primary antibodies including CD31-Biotin (eBioscience), CD34-Biotin (eBioscience), CD45-Biotin (eBioscience), CD24-PE (eBioscience), EpCAM-PE-Cy7 (BioLegend, San Diego, CA), and Sca-1-APC (eBioscience) were added to incubate cells. Biotin-conjugated antibodies were detected following incubation with streptavidin-APC-Cy7 (eBioscience). Dead cells were discriminated by 7- amino-actinomycin D staining (PharMingen, San Diego, CA). For cell surface marker staining of immune cells, we stained cells in staining buffer on ice for 45 min with combinations of the following fluorescent-labeled mAb (BD Biosciences) as follows: CD45 (30-F11), CD3e(145-2C11), CD4(RM4-5), CD8a(53-6.7), CD45R/B220(RA3-6B2), NK1.1(PK136), CD25(PC61). The stained cells were finally resuspended in 400 µL of HBSS plus and filtered through a 40-µm cell strainer (BD Biosciences, San Diego, CA) for sorting or analysis. Cell sorting was performed with a Cytomation MoFlo (Beckman Coulter, Inc., Brea, CA). Flow cytometry analysis was performed with a BD FACS Fortessa (BD Bioscience, San Jose, CA), and data were analyzed with FlowJo software (Tree Star, Inc., Ashland, OR). Nonstained lung cells with the same preparation were also analyzed as the negative control and reference for data analysis. Means and standard error of the mean (SEM) of independent experiments were calculated.

### RNA Sequencing and Data Analysis

RNA extraction was performed on primary human small airway epithelial cells (hSAEC) after CXCL10 treatment for 24 h, and then RNA sequencing was performed using the Illumina HiSeq2500 platform (Berry Genomics, Beijing). FastQC (v.0.11.2) was used to control the quality of RNA-seq reads. Bowtie2 (v.2.1.0) and Tophat2 (v.2.0.11) were used to map RNA-seq reads to the human genome (v.hg19). Cufflinks (v.2.2.1), Cuffmerge (v.2.2.1), and Cuffdiff (v.2.2.1) software were used to assemble transcription units, calculate gene expression levels (FPKM value), and identify genes differentially expressed between samples (https://datadryad.org/stash/share/oYavCmQzmrm17zshaSN8YuuD-LLTH-8E9bYv7RaK_Nc). Then, pathway and biological process enrichment analyses were performed on the Metacore (Clarivate Analytics) platform for these differential genes. The Gene Ontology (GO) processes (*P* < 0.05, enriching genes with change fold greater than 2) and pathways (*P* < 0.05, enriching genes with change fold greater than 1.5) in the clustering results were visualized by Cytoscape software (v.3.6.1).

### Western Blotting

Whole cell lysates were prepared with lysis buffer (RIPA lysis buffer with protease inhibitor cocktails) and boiled at 95°C for 10 min. The boiled lysates were analyzed by SDS-PAGE before transfer to nitrocellulose transfer membranes (Whatman Protran, Buckinghamshire, UK). The membranes were probed with primary antibodies, anti-p-TrkA, anti-TrkA, anti-P-JNK, anti-JNK, and anti-GAPDH antibodies, and the proteins were visualized using horseradish peroxidase (HRP)-conjugated antibodies and a chemiluminescent substrate, then exposed to film. The band density was calculated using Quantity One software and the intensity bars represent the relative protein quantification from two intensity scans for one experiment.

### Biolayer Interferometry Analysis

The FortéBio Octet Red96e instrument was used in this assay. The experiment consisted of three steps; *1*) baseline, *2*) association, and *3*) dissociation. Biotinylated TrkA (Human TrkA, MedChemExpress, Monmouth Junction, NJ; Biotinylation Kit, Genemore, JiangSu) was immobilized onto the streptavidin (SA) tip to probe the interaction between TrkA and CXCL10. Four concentrations (33, 100, 300, and 900 nM) of human CXCL10 (MedChemExpress, Monmouth Junction, NJ) were used for the association step. Conjugation and dissociation plots as well as kinetic constants were obtained using FortéBio data analysis software.

### Statistics

Differences in measured variables between genetically altered mice and the control group, or between treatment groups, were assessed using the Student’s *t* test or by one-way analysis of variance with Tukey–Kramer post hoc test (Prism; GraphPad, La Jolla, CA). Data are expressed as means ± SE where applicable. Statistical difference was accepted at *P* < 0.05.

## RESULTS

### CXCL10 Expression Is Induced in AEC2s by Lung Injury

**Figure 1.**
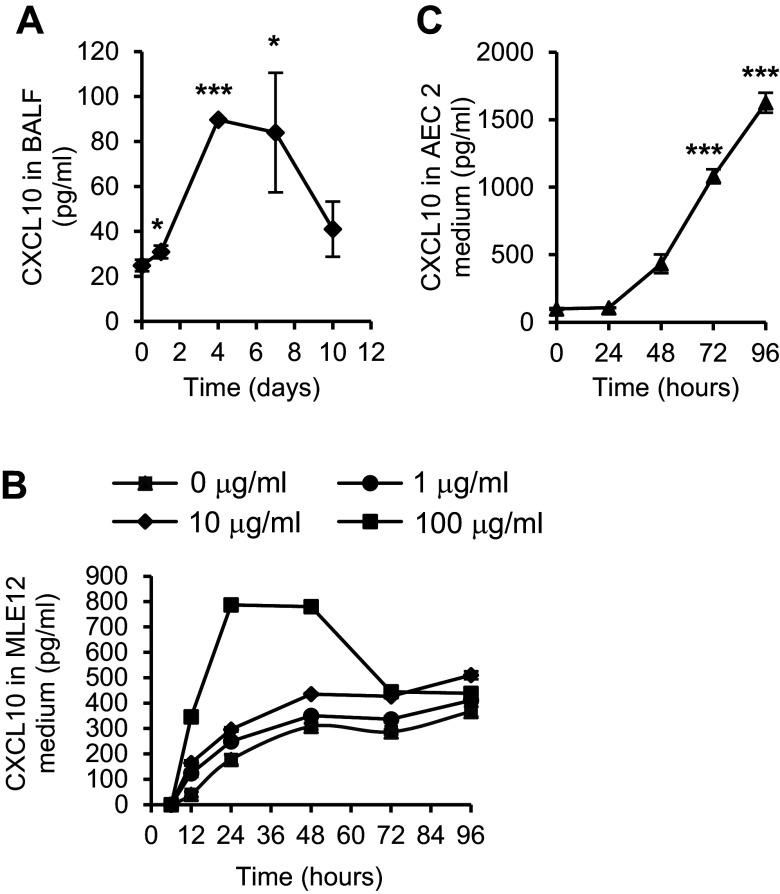
Bleomycin injury-induced C-X-C motif chemokine 10 (CXCL10) expression. *A*: CXCL10 was upregulated by bleomycin-induced lung injury. Bleomycin was given to C57BL/6 mice intratracheally, and bronchoalveolar lavage (BAL) was harvested 0, 1, 4, 7, and 10 days after injury. The CXCL10 levels in BAL were assessed by Bio-plex. *n* = 3–6 wild-type (WT) or Sftpc-Cxcl10 mice, *P < 0.05, *** *P* < 0.001, One-way ANOVA (Tukey’s test) and plotted as means ± SEM. *B*: CXCL10 expression was upregulated in MLE12 cells in a dose-/time-dependent manner after bleomycin treatment. *n* = 3 replicates. *C*: CXCL10 expression was upregulated in type 2 alveolar epithelial cells (AEC2s) injured in vivo by bleomycin. AEC2s were sorted from single-cell lung digestion 5 days after bleomycin treatment of WT mice and cultured for 96 h. *n* = 3 WT, 3 Sftpc-Cxcl10 mice, ***P < 0.001, One-way ANOVA (Tukey’s test) and plotted as means ± SEM.

CXCL10 can be expressed by lung epithelial cells (17), in addition to T cells and macrophages. To understand the role of CXCL10 in lung epithelial cells, we first examined CXCL10 expression from lung epithelial cells in response to injurious stimulus in vivo and in vitro. After treatment with bleomycin, there was a marked increase in CXCL10 expression in bronchioalveolar (BAL) fluid, from 25 pg/mL on *day 0* to a peak of 90 pg/mL on *day 4* (Fig. 1*A*). We also found that there was a significant increase in CXCL10 expression in the mouse lung epithelial cell line 12 (MLE12) after bleomycin treatment in culture (Fig. 1*B*). This increase was in a time- and dose-dependent manner, with a maximum CXCL10 expression at 24 h after bleomycin treatment.

We used a FACS-based strategy to purify lung AEC2s from single-cell homogenates stained with the antibodies to surface markers conjugated with fluorochromes (20). AEC2s were defined as EpCAM^+^Lin^–^CD24^–^Sca1^–^ (R4, Supplemental Fig. S2). We sorted AEC2 population from WT mice 5 days after bleomycin injury, cultured using normal two-dimensional method for 96 h, and detected the CXCL10 expression in the medium. The sorted mouse AEC2s showed upregulated CXCL10 in a time-dependent manner (Fig. 1*C*). These data are consistent with previous studies (17) and suggest that CXCL10 plays a role in the lung epithelial cell injury and repair.

### Generation of Sftpc-Cxcl10 Transgenic Mice

**Figure 2.**
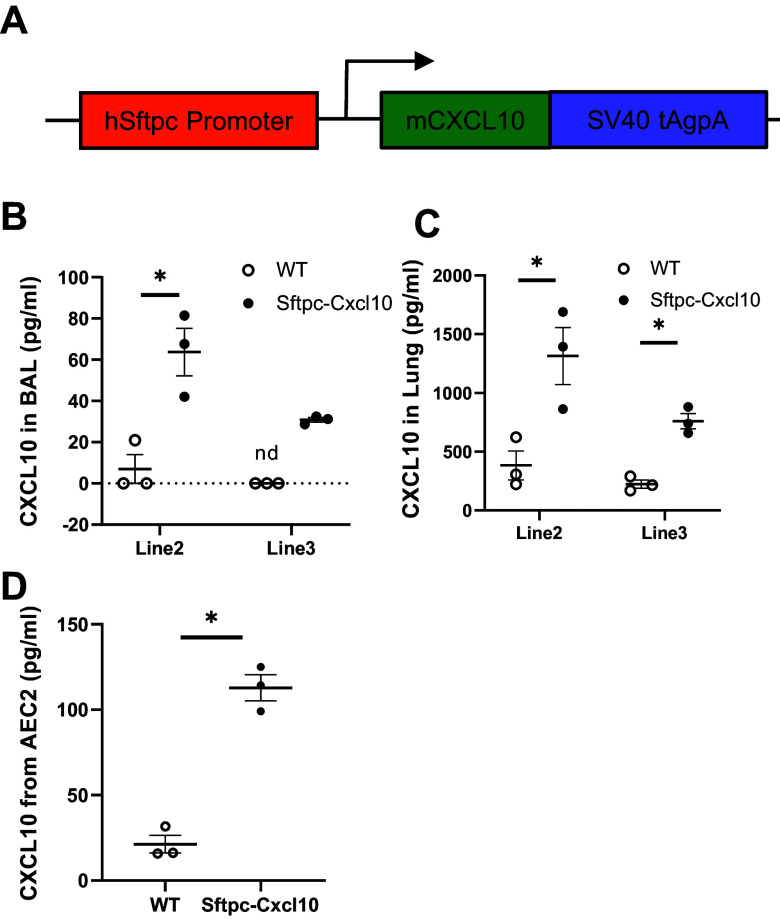
Overexpression of C-X-C motif chemokine 10 (CXCL10) in the lung type 2 alveolar epithelial cells (AEC2s). *A*: transgenic construct to show that the mouse CXCL10 cDNA was cloned downstream of the human Sftpc promoter and upstream of SV40 tAg polyadenylation. *B* and *C*: Sftpc-Cxcl10 transgenic mice produced a fair amount of CXCL10 into bronchoalveolar lavage (BAL) and lung tissue. *D*: culture of Sftpc-Cxcl10 AEC2s in three-dimensional (3-D) gel for 10 days produced more CXCL10 into colony medium compared with WT mice. CXCL10 levels in BAL(*B*), lung tissue in 8-wk-old transgenic mice and their WT littermates (*C*), and colony medium (*D*) were measured with ELISA (*n* = 3). n.d., not detected. *P < 0.05, Student’s *t* test and plotted as means ± SEM.

To assess the role of CXCL10 in vivo, we generated a new transgenic mouse line, Sftpc-Cxcl10, expressing CXCL10 specifically in Sftpc lineage alveolar epithelial cells. The mouse Cxcl10 cDNA was under the control of the human Sftpc (SPC) promoter (Fig. 2*A*). The transgenic mice were viable and fertile, with normal appearance and lung histology. The Sftpc-Cxcl10 mice produced moderate amounts of CXCL10 both in BAL (Fig. 2*B*) and lung tissue (Fig. 2*C*), compared with Scgb1a1-Cxcl10 transgenic mice (18).

We sorted AEC2 (EpCAM^+^Lin^–^CD24^–^Sca1^–^) population from the single-cell lung homogenates of untreated WT or Sftpc-Cxcl10 mice and cultured in 3-D gel for 10 days. The conditioned medium from cultured colonies of Sftpc-Cxcl10 AEC2s also showed a significant increase in CXCL10 expression compared with WT AEC2s (Fig. 2*D*).

### Overexpression of CXCL10 Prevents Mice from Bleomycin-Induced Lung Injury

**Figure 3.**
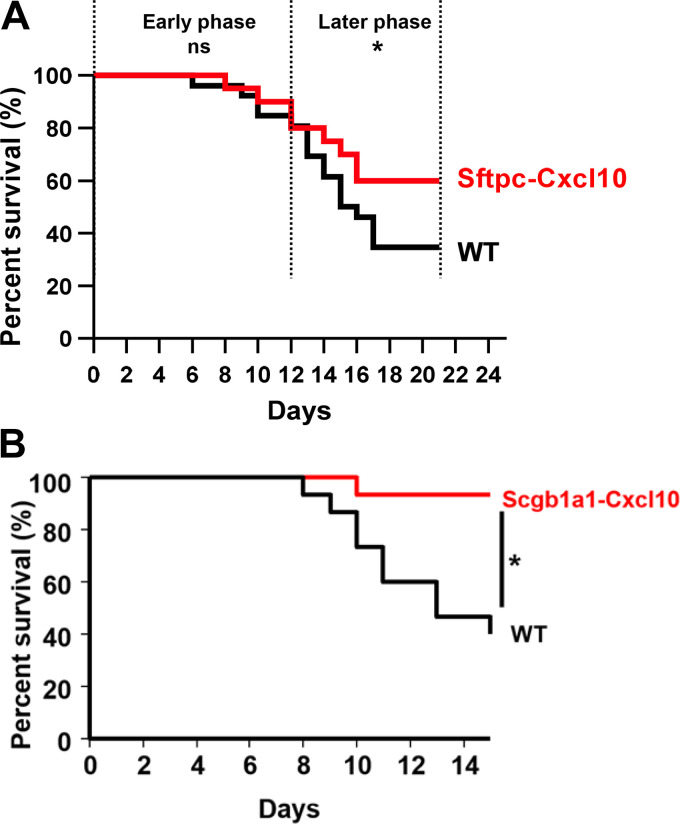
Increased survival rate was observed in Sftpc-Cxcl10 and Scgb1a1-Cxcl10 mice. Sftpc-Cxcl10 and littermate wild-type (WT) controls were subjected to bleomycin-induced lung injury (5 U/kg). *A*: the survival rate of Sftpc-Cxcl10 increased after bleomycin treatment during 21 days of observation (*n* = 10–15 WT, Sftpc-Cxcl10 mice). *B*: the survival rate of Scgb1a1-Cxcl10 increased after bleomycin treatment during 15 days of observation (*n* = 10–15 WT, Scgb1a1-Cxcl10 mice). Data are representative of three similar experiments. *P < 0.05. Simple survival analysis (Kaplan-Meier).

To examine the role of CXCL10 in lung injury and repair, bleomycin was instilled intratracheally into Sftpc-Cxcl10 mice and WT littermate controls. We observed that Sftpc-Cxcl10 mice were more resistant to bleomycin-induced lung injury, showing an increase in survival rate relative to WT mice (Fig. 3*A*). Intratracheal bleomycin instillation causes initial alveolar epithelial cell apoptosis with inflammatory cell infiltration in the early phase, and repair accompanied with fibrosis in the later phase (22, 23). Accordingly, we divided the survival curve into two phases. For the early phase (0–12 days), the survival rate of Sftpc-Cxcl10 mice after bleomycin remained the same as WT (80% on *day 12*, *P* = 0.99). On the contrary, for the later phase (13–21 days), the survival rate of Sftpc-Cxcl10 mice increased significantly compared with WT mice (60% for Sftpc-Cxcl10 vs. 34% for WT on *day 21*; *P* = 0.03, Fig. 3*A*). This protective effect was more pronounced as almost all of the Scgb1a1-Cxcl10 transgenic mice (mouse CXCL10 cDNA was cloned downstream of the rat Scgb1a1 promoter) (18) survived from bleomycin injury (Fig. 3*B*). These data suggest a key role for CXCL10 in protection of mouse epithelial injury. The difference of survival rate between these two strains of mice may be related to CXCL10 expression levels. Scgb1a1-Cxcl10 mice produce much more CXCL10 protein in the lung (18). However, the fibrosis did not decrease as indicated by trichrome staining (Supplemental Fig. S1*A*) and hydroxyproline content analysis (Supplemental Fig. S1*B*).

### Dysregulated Inflammation Both in Sftpc-Cxcl10 Mice after Bleomycin Lung Injury and Human Lung Cells Treated with CXCL10

**Figure 4.**
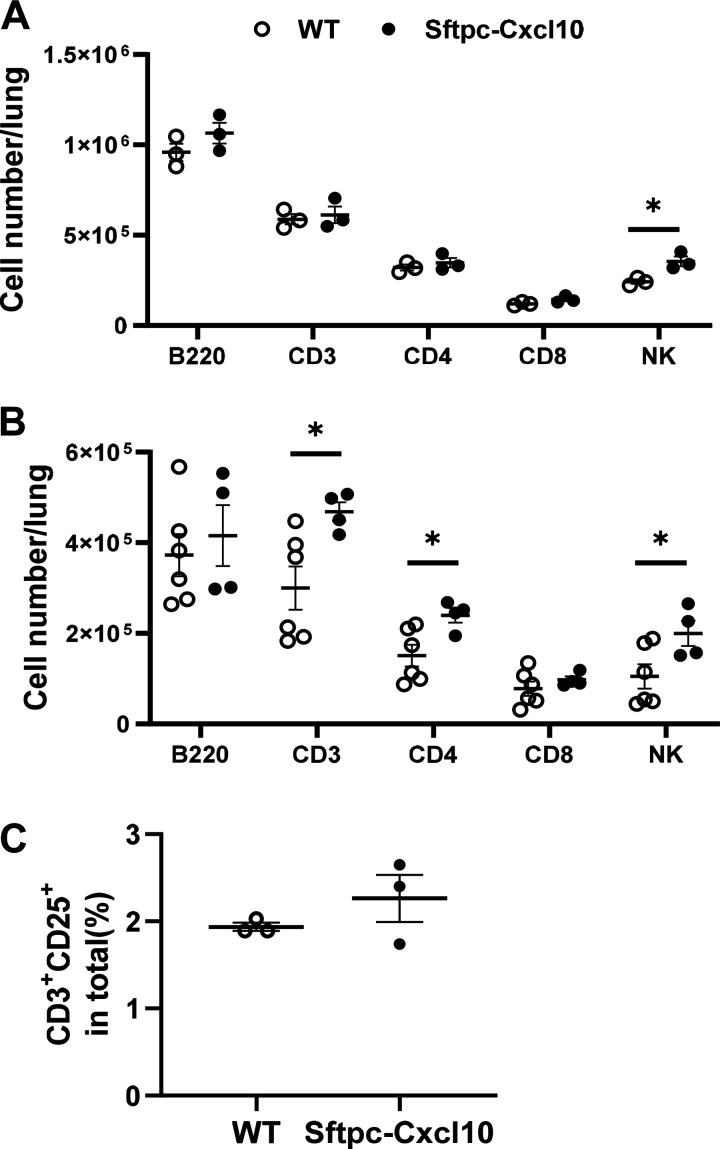
Dysregulated recruitment of inflammatory cells in Sftpc-Cxcl10 mice after bleomycin lung injury. After 7 or 10 days of bleomycin injury, single cells of lung were isolated and stained, and the immune cells were then analyzed with flow cytometry. Sftpc-Cxcl10 mice showed an increase in NK cells on *day 7* (*A*) and also an increase in total T cells and CD4^+^ T cells in lung single cell digestion on *day 10* (*B*) after bleomycin-induced lung injury. *n* = 4–6. *P < 0.05. *C*: activated T cells (CD3^+^CD25^+^) increased in Sftpc-Cxcl10 mice after 7 days of bleomycin injury. *n*=3-6 WT, Sftpc-Cxcl10 mice, Student’s *t* test and plotted as means ± SEM.

The bleomycin injury to the lung epithelium elicits an immune response. Damaged AEC2s release a variety of the chemokines that recruit inflammatory cells to the site of injury (24, 25). CXCL10 is known to recruit subsets of T cells (3). To investigate the mechanism by which overexpressed CXCL10 in AEC2s in the Sftpc-Cxcl10 transgenic mice regulate survival and fibrosis, we studied the CXCL10 effect on inflammation change in vivo. The inflammatory cell recruitment and activation in the Sftpc-Cxcl10 and WT mice during noninfectious acute lung injury were analyzed. Inflammatory cells increased dramatically in both mice after bleomycin injury in confirmation with previous data (19). We observed a marked increase in CD3^+^ T lymphocytes cells, CD4^+^ T and natural killer (NK) cells in the single-lung digestion in Sftpc-Cxcl10 mice compared with their WT littermates after 7 and 10 days of bleomycin treatment (Fig. 4, *A* and *B*). CD3^+^CD25^+^ cells are activated T-cell population. We also found an increasing tendency in this population in the Sftpc-Cxcl10 mice, suggesting that epithelial expression of CXCL10 recruits and activates CD3^+^ cells (Fig. 4*C*).

**Figure 5.**
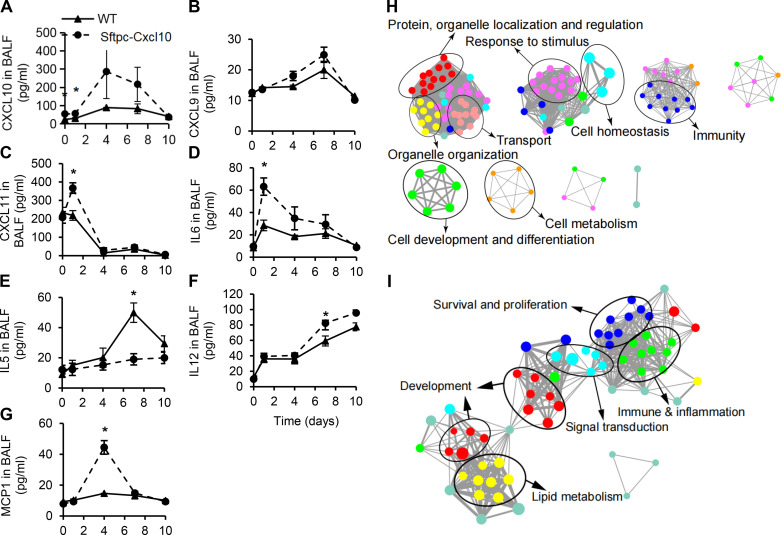
Dysregulated cytokine levels in Sftpc-Cxcl10 mice after bleomycin lung injury and enrichment analysis of differentially expressed genes (DEGs) in human small airway epithelial cells (hSAECs) treated with C-X-C motif chemokine 10 (CXCL10). After 0, 1, 4, 7, 10 days of bleomycin injury, bronchoalveolar lavages (BALs) were harvested and levels of CXCL10 (*A*), CXCL9 (*B*), CXCL11 (*C*), IL6 (*D*), IL5 (*E*), IL12 (*F*), monocyte chemoattractant protein (MCP)1 (*G*) were determined by Bio-plex or ELISA. hSAECs treated with CXCL10 for 24 h were used for RNA-seq and analysis. Functional processes and pathways from DEGs influenced by CXCL10 were enriched by Metacore. Biological process networks diagram (*H*) and pathway networks diagram of DEGs in the CXCL10 group (*I*) compared with the blank group (*P* < 0.05). The size of the node represents the number of DEGs in the gene set, the color of the node reflects the enrichment score [false discovery rate (FDR) value], and the edge line represents the overlap of DEGs. *P < 0.05 with their respective wild-type (WT) littermates. *n* = 3–6 WT, Sftpc-Cxcl10 mice, Student’s *t* test and plotted as means ± SEM.

Next we investigated the inflammatory cytokines in the BAL of Sftpc-Cxcl10 mice after bleomycin injury with Bio-plex and ELISA. Most of the cytokines measured showed an increase in WT mice after bleomycin injury. Furthermore, the increase of cytokines was more significant in Sftpc-Cxcl10 mice. We found CXCL9, CXCL10, CXCL11, IL12, IL6, IL5, and monocyte chemoattractant protein (MCP)1 changed in the Sftpc-Cxcl10 mice compared with WT, whereas we found no changes for IL2, IL4, IL10, IL13, and TNFβ (Fig. 5 and data not shown). CXCL9, CXCL10, and CXCL11 are ligands of CXCR3, and they were all increased in the BAL of Sftpc-Cxcl10 mice compared with WT (Fig. 5, *A–C*). IL6 was increased significantly in the *day 1* BAL and IL12 increased significantly in the *day 7* and *day 10* BAL for the Sftpc-Cxcl10 mice compared with WT (Fig. 5*D*). IL5 is an interleukin produced by Th2 cells and was significantly increased in the BAL of WT on *day 7* and *day 10*, but not in the Sftpc-Cxcl10 mice (Fig. 5*E*). MCP1 is a proinflammatory CC chemokine and it was significantly increased in *day 4* BAL of Sftpc-Cxcl10 mice compared with WT (Fig. 5*G*). No changes in active transforming growth factor (TGF)β were found (data not shown). These data suggested a dysregulated, robust inflammatory response bias to Th1 cytokines in the Sftpc-Cxcl10 mice.

We performed transcriptome analyses on human small airway epithelial cells (hSAECs) treated with human CXCL10 and vehicle control. A total of 488 differentially expressed genes (DEGs) were identified. The biological process enrichment analysis of DEGs showed significant changes in protein, organelle localization and regulation, organelle organization, transport, response to stimulus, immunity, cell development, differentiation, and metabolism after CXCL10 treatment for 24 h compared with control (Fig. 5*H*). Pathway enrichment analysis of DEGs revealed significant changes in pathways related to survival and proliferation, immune and inflammation, signal transduction, lipid metabolism, and development (Fig. 5*I*). The altered inflammatory and immune response in hSAECs is consistent with the inflammation dysregulation found in Sftpc-Cxcl10 mice.

### Sftpc-Cxcl10 Transgenic Mice Exhibit Increased AEC2s after Lung Injury

**Figure 6.**
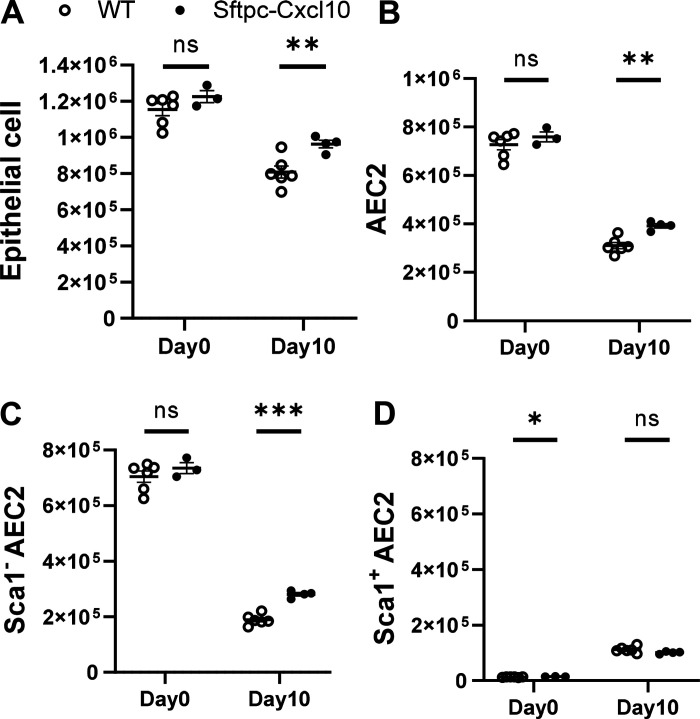
Boosted regeneration of Sftpc-Cxcl10 AEC2s in vivo after bleomycin injury. Bleomycin was given to Sftpc-Cxcl10 mice and wild-type (WT) mice intratracheally, lungs were harvested 0 and 10 days after injury, and single cells were isolated by Elastase and DNase I digestions. Type 2 alveolar epithelial cells (AEC2s) were determined by staining cells with specific antibodies. The cell number of epithelial cells (EpCAM^+^Lin^–^) (*A*), AEC2s (*B*), Sca1^–^ (*C*), and Sca1^+^ AEC2s (*D*) were calculated by multiplying the total cell number of each lung and the percentage of each specific cell group in total cells (*n* = 6 WT,3-4 Sftpc-Cxcl10 mice). *P < 0.05, **P < 0.01, ***P < 0.001. Student’s *t* test and plotted as means ± SEM.

To further investigate the mechanism of lung injury in Sftpc-Cxcl10 transgenic mice, we studied the CXCL10 effect in vivo on the AEC2 regeneration efficiency. After bleomycin injury, the epithelial cells are damaged in the early phase, release a variety of the chemokines that help form the regenerative niche, and will gradually recover in the later phase (22, 23). The EpCAM^+^Lin^–^ epithelial cell number decreased after bleomycin injury in both Sftpc-Cxcl10 and WT mice on all the days we checked (*days 4*, *7*, and *10*) (Fig. 6*A* and data not shown). However, we found significantly increased EpCAM^+^Lin^–^ epithelial cell population in the lung single-cell digestion in the Sftpc-Cxcl10 transgenic mice compared with WT mice 10 days after bleomycin injury, with only a tendency of increase on *days 4* and *7*. Examination of the EpCAM^+^Lin^–^CD24^–^ AEC2 population revealed an increase in Sftpc-Cxcl10 transgenic mice compared with WT littermates, too (Fig. 6*B*).

We further analyzed the Sca1^–^ population in the EpCAM^+^Lin^–^CD24^–^ AEC2s group (R5, Supplemental Fig. S1). We found a significant increase in Sca1^–^AEC2s (Fig. 6*C*), in Sftpc-Cxcl10 mice compared with WT. These data imply that the Sca1^–^ AEC2s contribute majorly to the increase in the total AEC2s number in Sftpc-Cxcl10 mice lung. All of the *day 0* data show no difference in the populations of epithelial cells or the AEC2 subsets between WT and Sftpc-Cxcl10 mice (Fig. 6, *A–D*), suggesting that the CXCL10 expressed by AEC2s mainly effects during the damage-induced regeneration, whereas conceal its function during the steady state. Altogether, these data indicate an enhanced regeneration of AEC2s from bleomycin-induced lung injury in the Sftpc-Cxcl10 mice, which might affect the survival rate and the extent of fibrosis.

### Augmented Regeneration Ability for Sftpc-Cxcl10 AEC2s

**Figure 7.**
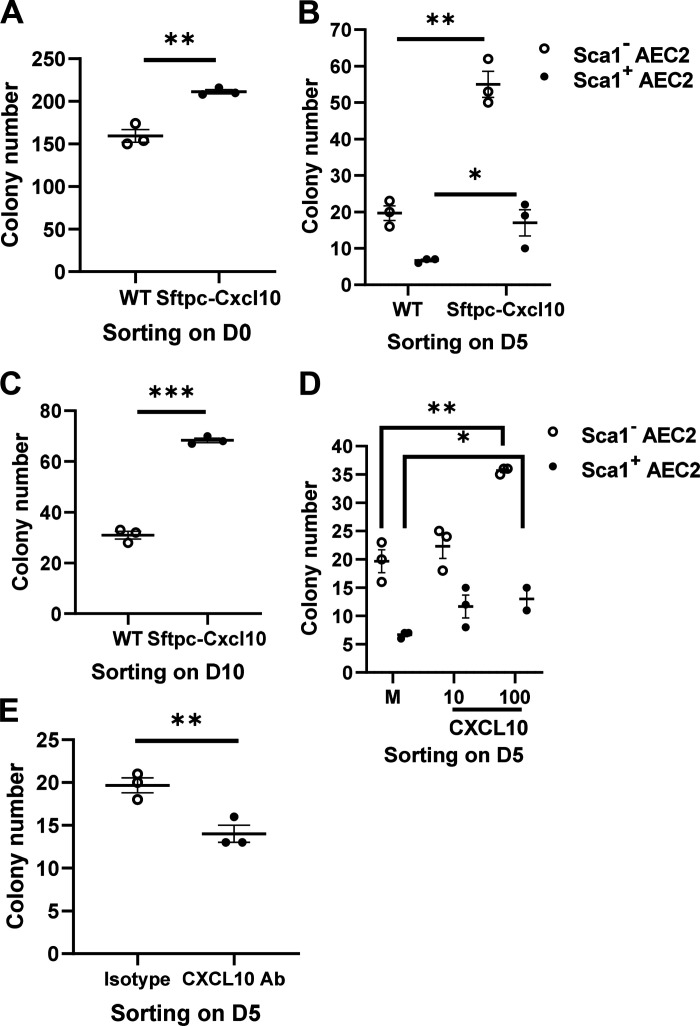
Regulation of colony formation efficiency (CFE) of type 2 alveolar epithelial cells (AEC2s) by C-X-C motif chemokine 10 (CXCL10). After 0, 5, and 10 days of bleomycin injury, single cells of lung were isolated, then small airway epithelial cells (SAEC2s) were sorted and CFE was determined. *A*–*C*: compared to the wild-type (WT) control, Sftpc-Cxcl10 mice showed significantly increased CFE of Sca1^−^ AEC2s sorted on *day 0* (*A*), *day 5* (*B*), or *day 10* (*C*) post-bleomycin treatment. Recombinant CXCL10 at 100 ng/mL showed an increase (*D*), and neutralizing antibody against CXCL10 at 10 µg/mL showed a decrease in CFE of WT Sca1^−^ AEC2s sorted on *day 5* (*E*) post-bleomycin compared with the medium (M) or the isotype control. *P < 0.05, **P < 0.01, ***P < 0.001. *n* = 3 WT, Sftpc-Cxcl10 mice for *A*–*C*, *n* = 2–3 replicates for *D*-*E*, Student’s *t* test for *A*-*C*, *E*, One-way ANOVA (Tukey’s test) for *D*, plotted as means ± SEM.

Typically, stem cells provide a rich source of cytokines and growth factors that can act in an autocrine, paracrine, or endocrine fashion to regulate cell behavior postinjury. To test the role of CXCL10 secreted from AEC2s as shown in Figs. 1*C* and 2*D*, and to elucidate the mechanism underlying the increased AEC2s population in the Sftpc-Cxcl10 transgenic mice compared with WT mice after injury (Fig. 6, *A–C*), we went further to detect the proliferative ability of Sftpc-Cxcl10 AEC2s using colony formation assay in vitro (20). AEC2s were sorted on 0, 5, or 10 days after bleomycin treatment, plated in 50% matrigel in the presence of MLg2908 fibroblasts. The colonies were normal in their morphology and epithelial marker expression patterns as described in previous studies (9, 20) (Supplemental Fig. S1*B*). There were 30% more colonies formed by Sca1^−^ AEC2s sorted from untreated (*day 0*) Sftpc-Cxcl10 mice compared with WT mice (Fig. 7*A*). When AEC2s were sorted on *day 5* after bleomycin, the colony formation efficiency (CFE) of AEC2s from Sftpc-Cxcl10 was even much higher than that of WT AEC2s, with 120% more colonies formed by Sca1^−^ AEC2s and 280% more colonies formed by Sca1^+^ AEC2s (Fig. 7*B*). This increase was also evident with Sca1^−^ AEC2s from *day 10* post-bleomycin, with 110% more colonies formed by Sca1^−^ AEC2s (Fig. 7*C*). Note Sca1^+^ AEC2s only emerge in the early-phase post-bleomycin and disappear in the later phase.

We next examined whether exogenous CXCL10 protein could enhance CFE of AEC2s sorted from lung homogenates of WT mice 5 days after bleomycin treatment. A significant increase in CFE was observed when recombinant CXCL10 protein was added in 3-D culture (Fig. 7*D*). Anti-CXCL10 neutralizing antibodies were then used to block the effect of CXCL10 on WT AEC2s 5 days post-bleomycin. The CFEs were significantly reduced compared with the isotype control (Fig. 7*E*).

These data suggest that the CXCL10 expressed from AEC2s could work as mitogen on AEC2s themselves in an autocrine manner. The higher regenerative capacity of Sftpc-Cxcl10 AEC2s sorted from lungs 5 days post-bleomycin compared with *day 0* and *day 10* suggests that Sftpc-Cxcl10 AEC2s were somehow activated by injury and hence might be uniquely responsive to the regenerative signaling from CXCL10.

### CXCL10 Proliferative Effect on Murine Epithelial Cells Is CXCR3 Independent

**Figure 8.**
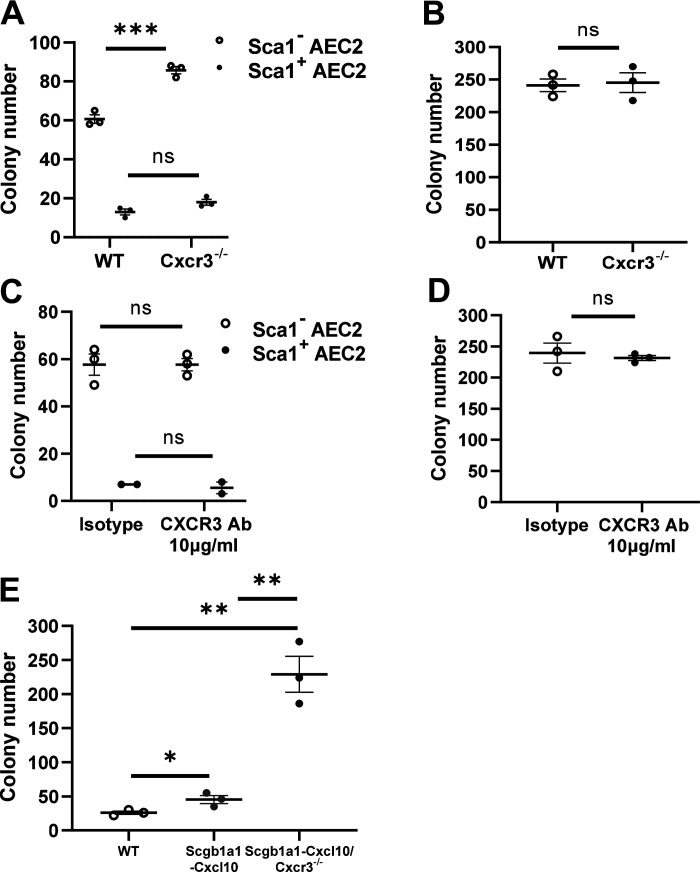
Effect of C-X-C motif chemokine 10 (CXCL10) on type 2 alveolar epithelial cells (AEC2s) is CXCR3 independent. AEC2s cells were isolated from lungs of wild-type (WT) or CXCR3 knockout (KO) mice 5 days (*A* and *C*) or 0 day post-bleomycin (*B* and *D*), then cultured without(*A* and *B*) or with (*C* and *D*) CXCR3 antibody at 10 µg/mL. *E*: AEC2s were isolated from lungs of WT, Scgb1a1-Cxcl10 mice, and Scgb1a1-Cxcl10-Cxcr3^−/−^ mice. Colony numbers were analyzed after 10–14 days of three-dimensional (3-D) gel culture. *P < 0.05, **P < 0.01, ***P < 0.001. *n* = 3 WT, 3 Cxcr3^-^/^-^ mice for *A*-*B*, *n* = 2–3 replicates for *C*-*D*, *n* = 3 WT, 3 Scgb1a1-Cxcl10 mice, and 3 Scgb1a1-Cxcl10-Cxcr3^-^/^-^ mice for *E*, Student’s *t* test for *A*–*D*, One-way ANOVA (Tukey’s test) for *E*, plotted as means ± SEM.

Effects of CXCL10 on chemoattraction of hematopoietic cells are mostly dependent on CXCR3 (2, 3, 26). However, CXCR3-independent pathways are found playing important roles in nonhematopoietic cells not expressing CXCR3 (4, 27). Our previous data also confirmed an antifibrotic function of CXCL10, binding to syndecan-4, instead of CXCR3, on fibroblasts (5). To directly test whether the proliferative effect of CXCL10 on AEC2s requires CXCR3, we isolated AEC2s on *day 0* and *day 5* after bleomycin treatment of the WT and *Cxcr3-*deficient mice, or cultured isolated WT AEC2s in 3-D gel with or without an anti-CXCR3 antibody. The colony formation assay showed an unexpected increase in the colony number of *Cxcr3-* deficient AEC2s compared with that of WT sorted on *day 5* (Fig. 8*A*), whereas no change was detected on *day 0* (Fig. 8*B*). Similarly, CXCR3 antibody also showed no effects on the CFE of WT AEC2s sorted on *day 5* and *day 0* (Fig. 8, *C* and *D*). As have been shown in Fig. 1*C*, the AEC2s sorted 5 days after bleomycin secreted high level of CXCL10. The proliferative effect of CXCL10 on epithelial cells did not abolish in the CXCR3 antibody group (Fig. 8*C*), while actually enhanced in the *Cxcr3-*deficient mice (Fig. 8*A*). We utilized the double modified Scgb1a1-Cxcl10-Cxcr3^−/−^ mice to confirm this. Airway progenitor cells were isolated from the lungs according to a flow cytometry-based method as previously described (20). The Scgb1a1-Cxcl10-Cxcr3^−/−^ colonies dramatically increased compared with the Scgb1a1-Cxcl10 group (Fig. 8*E*). Appraisal of the data shows that during the process of AEC2 injury and repair induced by bleomycin, CXCR3 seems to be antiproliferative and that the proliferative effect of CXCL10 on lung epithelial cells might be independent of CXCR3, in consistence with the CXCR3 independent effects of CXCL10 in other nonhematopoietic cells including endothelial cells and fibroblast (4, 5, 27).

### The TrkA Pathway May Participate in the Proliferative Effect of CXCL10 on Murine Epithelial Cells

**Figure 9.**
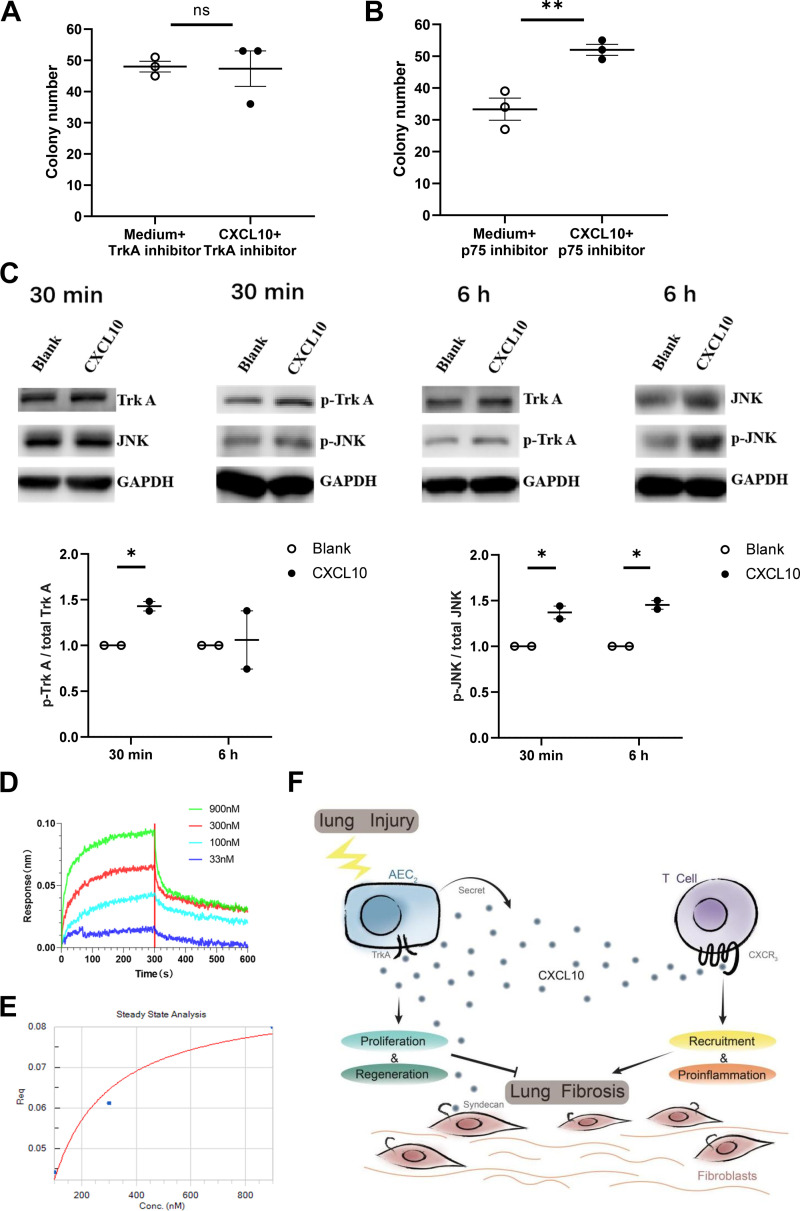
C-X-C motif chemokine 10 (CXCL10) regulates type 2 alveolar epithelial cell (AEC2) renewal through TrkA. AEC2s cells were isolated from lungs of wild-type (WT) mice 5 days post-bleomycin. TrkA inhibitor (*A*) or p75 inhibitor (*B*) were added to AEC2 cells with or without CXCL10, respectively. Colony numbers were analyzed after 10–14 days of three-dimensional (3-D) gel culture. *C*: Western blotting analysis of p-TrkA and p-JNK in A549 cells treated with CXCL10 for 30 min or 6 h. The blots were analyzed with anti-p-TrkA, anti-TrkA, anti-p-JNK, anti-JNK, and anti-GAPDH antibodies. p-TrkA, phosphorylated TrkA; p-JNK, phosphorylated JNK. Untreated A549 cells served as a blank control.*D*: simulated binding curves of different concentrations of CXCL10 to immobilized TrkA. *E*: steady-state analysis of the binding curves of CXCL10 (from 33 nM to 900 nM) to immobilized TrkA. *F*: a schematic showing that CXCL10 may recruit T cells through CXCR3, while promote regeneration of AEC2s through TrkA. *P < 0.05, **P < 0.01. *n* = 3 replicates for *A*–*B*, *n*=2 replicates for *C*, Student’s *t* test for *A*–*C*, plotted as means ± SEM.

During the virus infectious lung injury, the lung epithelial cells experience pathological changes from injury to regeneration. A previous study found that NGF that normally controls the survival, proliferation, and growth of neuronal cells, could also promote lung epithelial cells to survive during respiratory syncytial virus infection, with overexpressed NGF and its high-affinity receptor TrkA and concomitant downregulation of the low-affinity receptor p75 (28). Thus, we used TrkA and p75 inhibitors to block their effects on sorted AEC. TrkA inhibitor eliminated the CFEs of CXCL10 as revealed in Fig. 7*D* (Fig. 9*A*), but p75 inhibitor did not (Fig. 9*B*). TrkA is a member of Receptor Tyrosine Kinase family, which can activate the classical RTK-RAS-MAPK pathway. We further checked the phosphorylation state of TrkA and JNK in A549 cells (human airway epithelial cells) treated with CXCL10, which indicates the activating state of the TrkA pathway. The ratio of pTrkA/TrkA and pJNK/JNK both increased significantly after treatment with CXCL10 for 30 min whereas the pJNK/JNK ratio remained significantly higher at 6 h than control (Fig. 9*C*). We further confirmed the binding of CXCL10 to TrkA by BLI assay (Fig. 9, *D* and *E*). Using different concentrations (33, 100, 300, and 900 nM) of CXCL10 bound to immobilized TrkA, and with the probe-based biosensor for direct sample detection and real-time monitoring of the binding process, we obtained the binding/dissociation binding curves (Fig. 9*D*). We then performed steady-state analysis using FortéBio data analysis software (Fig. 9*E*), and analyzed the system by fitting calculations to obtain an affinity (*K*_D_) value of 51.3 nM. These results indicate that CXCL10 interacts with TrkA directly and reversibly.

Altogether, the data presented herein suggest that CXCL10 can exert not only the paracrine signaling to recruit T cells, but also the autocrine signaling involved in the regeneration of AEC2s, although with different receptors and leading to different cell responses (Fig. 9*D*).

## DISCUSSION

The adult lung is normally nonproliferative, with prolonged survival of resident AEC2s and AEC1s maintaining the function for gas exchange. Upon loss or damage to these cells, they are replaced by the regeneration and differentiation of AEC2 stem/progenitor cells, which constitute an important part for recovery from lung injury (9, 29). How this repair is orchestrated by chemokines in the epithelium microenvironment is unclear. At the beginning of its discovery, CXCL10 has been classified to a family of chemotactic and mitogenic proteins based on the homology of primary structure, which are related with inflammation and proliferation (30). The inflammation role of CXCL10 on hemopoietic cell through CXCR3 receptor is extensively studied in many disease models already (18, 31–33). However, the effects of CXCL10 on epithelial cells have rarely been studied in vitro or in vivo. We therefore focused our study on the rarely-studied effect of CXCL10 on injury and repair of AEC2s, using a newly generated Sftpc-Cxcl10 transgenic mice that overexpress CXCL10 in AEC2s and a 3-D culture assay. We found a specialized proliferation ability of CXCL10 on the AEC2s independent of CXCR3 in an autocrine manner.

AEC2s are the most numerous epithelial cell lining the alveoli and are susceptible to external irritants such as inflammation, infection, or physiochemical material exposure. These irritants impair functions of AEC2s, including regeneration and re-epithelialization after alveolar damage, leading to declined lung function and further fibrosis (34–36). In many infectious models, the virus target and damage AEC2s directly and induce lung injury (34, 35). In IPF, loss or damage to AEC2s is important early pathological features for the initiation of the disease and insufficient repair of AEC2s contributes to further fibrosis progression (36, 37). We showed that there is a loss of AEC2s, and a failure of AEC2 renewal in IPF (10). Bleomycin-induced lung injury model is a widely accepted noninfectious model for the study of lung injury, repair, and fibrosis. In this study, intratracheal instilled bleomycin decreased the cell number of the AEC2 population remarkably for all the time points we have checked, including *day 10*, compared with nontreated mice. Moreover, the sorted AEC2s after bleomycin treatment also presented dramatically decreased CFE compared with the AEC2s sorted on *day 0*.

It is critical that the site of lung injury is quickly closed and replaced by newly differentiated epithelial cells to maintain an effective physical barrier and improve survival. Understanding the mechanisms and modulators that regulate the repair of AEC2s has great significance for lung injury therapy. Wnt (38), hypoxia-inducible factor 1 α (HIF-1a) (39), and fibroblast growth factor 2 (Fgfr2) signaling (40) pathways have been recently found important for the regenerative capacity of AEC2s. Palifermin, a FDA approved anti-mucositis drug, is a keratinocyte growth factor and found to promote epithelial survival, both in animal model and in a human lung injury model induced by inhaled LPS, supposing beneficial effects in epithelial survival and proliferation in acute respiratory distress syndrome (ARDS) (41). CXCL10 was found in our study to promote AEC2 regeneration in vivo and in vitro. In the early phase featuring apoptosis and inflammation, such as on *day 5* after bleomycin injury, we found no significant change in the total AEC2s population in the Sftpc-Cxcl10 mice compared with WT mice (data not shown). However, in the later phase featuring regeneration and fibrosis, i.e., on *day 10* post-bleomycin, the Sftpc-Cxcl10 mice presented noteworthy stronger ability of AEC2s regeneration from injury. We found more epithelial cells (R3, EpCAM^+^Lin^–^), AEC2s (R4, EpCAM^+^Lin^–^CD24^–^), and Sca1^–^ AEC2s (R5, EpCAM^+^Lin^–^CD24^–^Sca1^–^) on *day 10* post-bleomycin in the single-cell digestion of the lungs of Sftpc-Cxcl10 mice compared with WT mice. This increased regeneration of AEC2s in vivo may result from the autocrine action of overexpressed CXCL10 from Sftpc-Cxcl10 AEC2s onto AEC2s themselves, which is confirmed by the following 3-D gel culture studies in vitro. Firsty, the AEC2s from Sftpc-Cxcl10 transgenic mice exhibited significantly increased colony number compared with that of WT mice, both in nontreated and bleomycin-treated conditions. Furthermore, albeit to a lesser degree, the addition of exogenous CXCL10 recombinant protein behaves similarly to endogenous CXCL10 from Sftpc-Cxcl10 AEC2s, further confirming the proliferation ability of CXCL10 on AEC2s. Finally, the CFE of AEC2s could be inhibited by addition of mouse CXCL10 antibody, while not by the isotype control antibody, which reliably confirms the effect of CXCL10 on AEC2s from the reverse side. Thus, we elucidated solidly that CXCL10 plays an important role in the proliferation of AEC2s using an autocrine signaling manner. During lung injury, the damaged AEC2s are induced to express CXCL10, a mitogenic factor to which they can also respond. Consequently, the cells bathe themselves continuously in CXCL10 that stimulates them to grow. In the case of the Sftpc-Cxcl10 transgenic mice, their AEC2s overexpressed CXCL10 and exhibited an enhanced proliferation ability in vitro. This deduction provided a pretty good explanation for the better survival rate found in the Sftpc-Cxcl10, especially in the later phase (*days 12*–*21*), which came after the time that Sftpc-Cxcl10 AEC2s began to show increased number compared with WT mice in vivo. Therefore, in Sftpc-Cxcl10 mice, the phase-dependent change in AEC2s population in vivo corresponded to the changes in survival rate after bleomycin injury. It is of interest that Sca1^+^ cells emerge during early injury. We do not know whether these cells are related to recently identified transitional state of AEC2s (42–45) or MHC^high^ club-like cells (46). We recently reported that MHC class II genes such as H2-K1, H2-D1, and H2-Q7 were upregulated in AEC2s from old mice (47). One would expect some overlaps exist between these populations. Further lineage tracing experiments are needed to determine the sources and functions of these populations. Taken together, we show here fortified regeneration and repair of AEC2s in Sftpc-Cxcl10 mice, which plays an important role in improving the survival rate.

The clinical diseases associated with pulmonary fibrosis are essentially classified into two distinct phenotypes of fibrosis. The first phenotype is postinflammatory fibrosis as shown in hypersensitivity pneumonitis (HP) and the second phenotype is dysregulated matrix deposition observed in idiopathic pulmonary fibrosis (IPF) (37). *1*) HP recapitulates a robust inflammatory associated with infiltrating activated T cells and IFNγ-inducible chemokines. We detected increased expression of endogenous CXCL10 in the Sftpc-Cxcl10 mice, as well as other IFNγ-inducible chemokines such as the monokine induced by IFNγ (MIG, CXCL9) and IFNγ-inducible T cell α chemoattractant (I-TAC, CXCL11). The dysregulated cytokines in the BAL and immune cells attracted in the lung of Sftpc-Cxcl10 mice showed evidence of Th1 cell involvement in the pathogenesis of postinflammatory fibrosis, relating to the CXCR3-dependent recruiting of T lymphocytes by CXCL10. *2*) The second phenotype of fibrosis in IPF involves mechanisms combining continuous injuries to the lung with inadequate repair of the epithelium (37). The repair and regeneration ability of AEC2s is extremely important for the progression and final outcome of lung fibrosis. The improved CXCR3-independent regeneration of AEC2s and enhanced repair of the epithelium by CXCL10 in the Sftpc-Cxcl10 would thus ameliorate fibrosis of this phenotype. Thus, the two distinct mechanisms of fibrosis are closely related with the two different functions of CXCL10, the proinflammation effect of CXCL10 on immune cells and the proliferative effect of CXCL10 on AEC2s, in the same model. In other words, the significantly stronger inflammation in the Sftpc-Cxcl10 mice induce increased tendency to the postinflammatory fibrosis, whereas the enhanced regeneration of AEC2s might hold back fibrosis. Thus, CXCL10 seems to play a role of double-edged sword in the fibrosis pathogenesis of the Sftpc-Cxcl10 mice. The proliferative effect on AEC2s counterbalances the proinflammation effect on immune cells. As a result, we did not find decreased hydroxyproline level in Sftpc-Cxcl10 mice as expected from the beneficial effect of CXCL10 on AEC2s, although we indeed observed an increased survival rate for the Sftpc-Cxcl10 mice in the later phase. Current study also supports our hypothesis in a previous paper that a mutant CXCL10, without CXCR3-binding activity, delivered directly to the lungs could be a novel therapeutic agent in pulmonary fibrosis (5). Eliminating CXCR3 binding could avoid potential toxicities related to T cell activation while preserving the proliferation ability of CXCL10 on AEC2s.

CXCL10 exerts its chemoattractic function for inflammatory cells through CXCR3-A. Some groups claimed that the isoform CXCR3-B played an important role for the function of CXCL10 on the nonhematopoietic cell, such as endothelial cells (48). However, the existence of a functional CXCR3-B transcript is still in debate (4). A study suggested that epithelial and endothelial cells express a functional and CXCL10-specific receptor that is neither CXCR3 nor glycosaminoglycan (49).

Our present study suggests that the regenerative effect of CXCL10 on AEC2s is independent of CXCR3. We further show that TrkA was activated by CXCL10 treatment and involved in the regenerative effect of CXCL10 on AEC2s. The colony formation assay using TrkA inhibitor abolished the regenerative effect of CXCL10 on AEC2s, whereas another inhibitor against the high affinity NGF receptor, p75, cannot. Furthermore, binding experiments indicate that CXCL10 interacts with TrkA directly and reversibly. Nevertheless, more experiments are needed to verify whether TrkA is a bona fide binding partner for CXCL10 on AEC2s, such as co-immunoprecipitation, mutational analysis, as well as genetic deletion. One would imagine that the CXCL10, with different functional domains, will bind different receptors on different cell types, and exert different functions. Further study is needed for the clarification of the different functional domains of CXCL10 and different receptors.

Taken together, we present evidence that the chemokine CXCL10 promotes the regeneration of AEC2s stem/progenitor cells both in vivo and in vitro. The ability of CXCL10 to promote AEC2s proliferation might occur independent of the CXCR3 receptor and requires probably TrkA. Further studies exploring the regenerative functions of CXCL10 on AEC2s could lead to new therapeutic options for both infectious and noninfectious lung injury.

## DATA AVAILABILITY

All data are available in the main text or the supplemental material.

## SUPPLEMENTAL MATERIAL

10.6084/m9.figshare.25688688 Supplemental Figs. S1 and S2: https://doi.org/10.6084/m9.figshare.25688688.

## GRANTS

This work was supported by National Natural Science Foundation of China Grant 81570077 (to Y.Z.) and by National Institutes of Health Grant P01HL108793 (to D.J. and P.W.N.).

## DISCLOSURES

No conflicts of interest, financial or otherwise, are declared by the authors.

## AUTHOR CONTRIBUTIONS

P.W.N. and D.J. conceived and designed research; Y.Z., J.Y., and N.L. performed experiments; Y.Z. and J.Y. analyzed data; Y.Z., J.L., and D.J. interpreted results of experiments; Y.Z. and J.Y. prepared figures; Y.Z. drafted manuscript; Y.Z. and D.J. edited and revised manuscript; Y.Z. and D.J. approved final version of manuscript.
